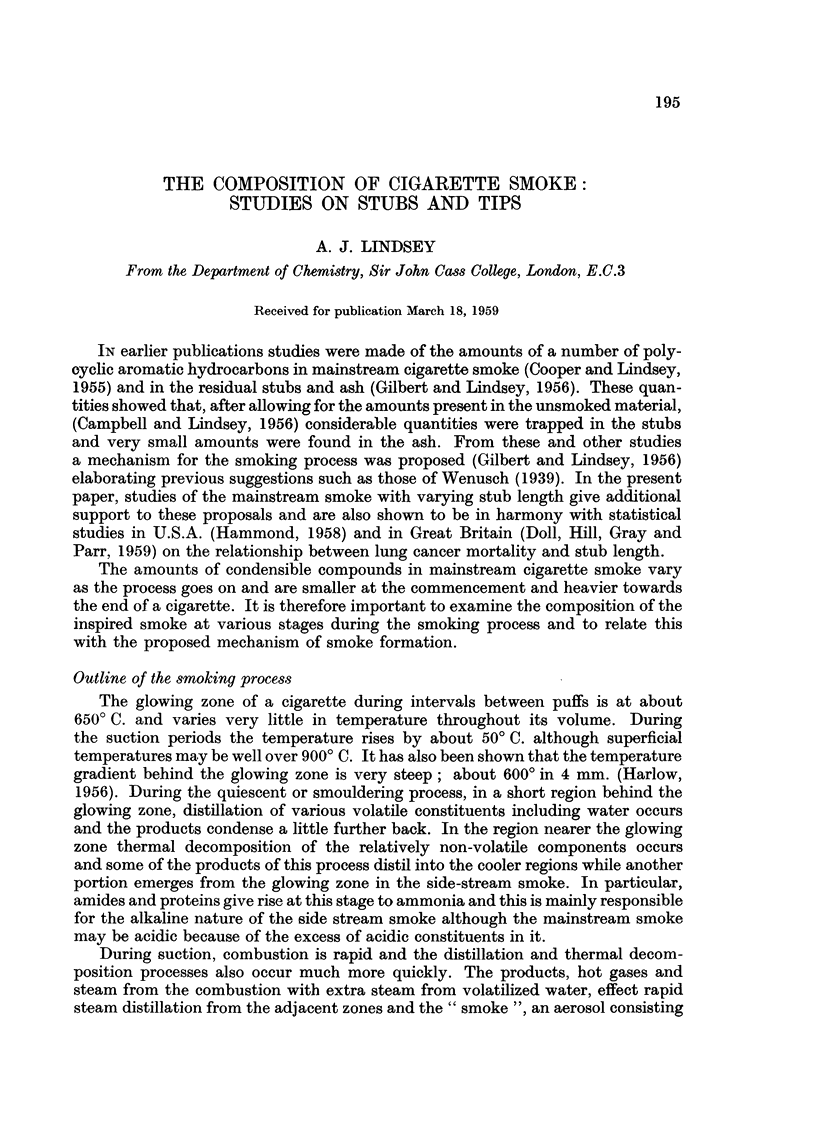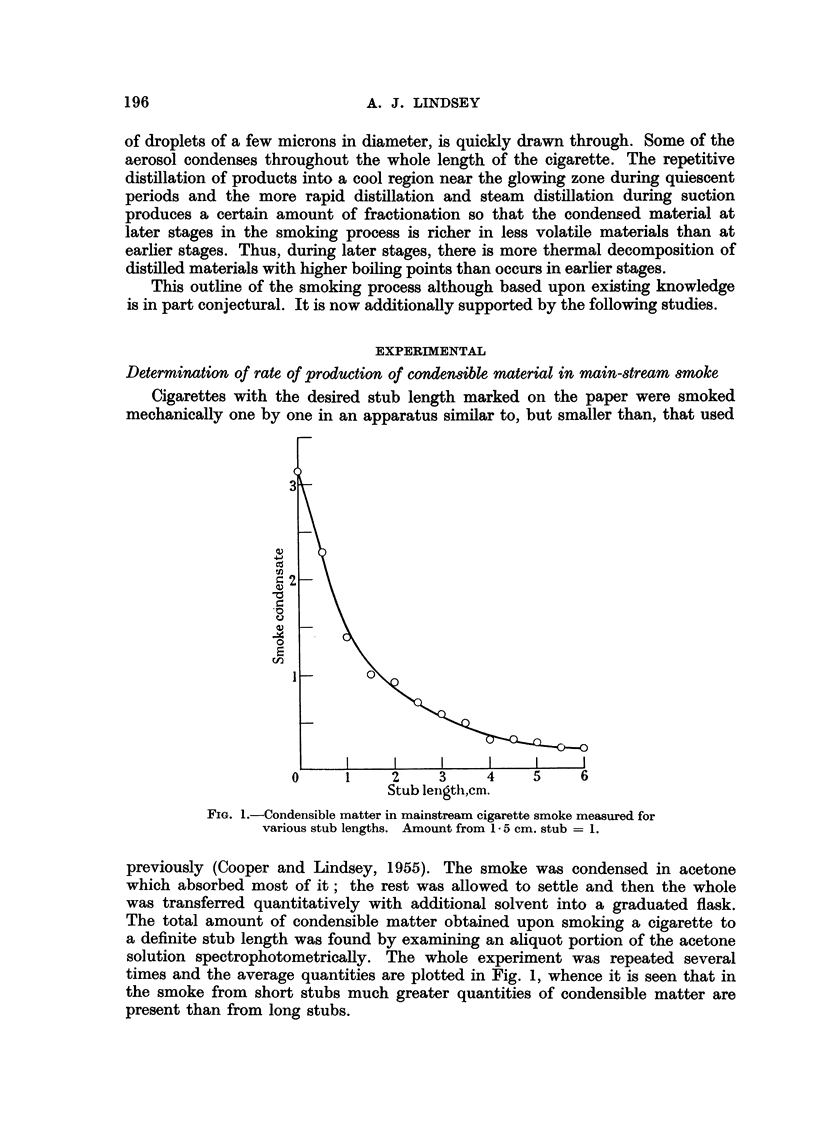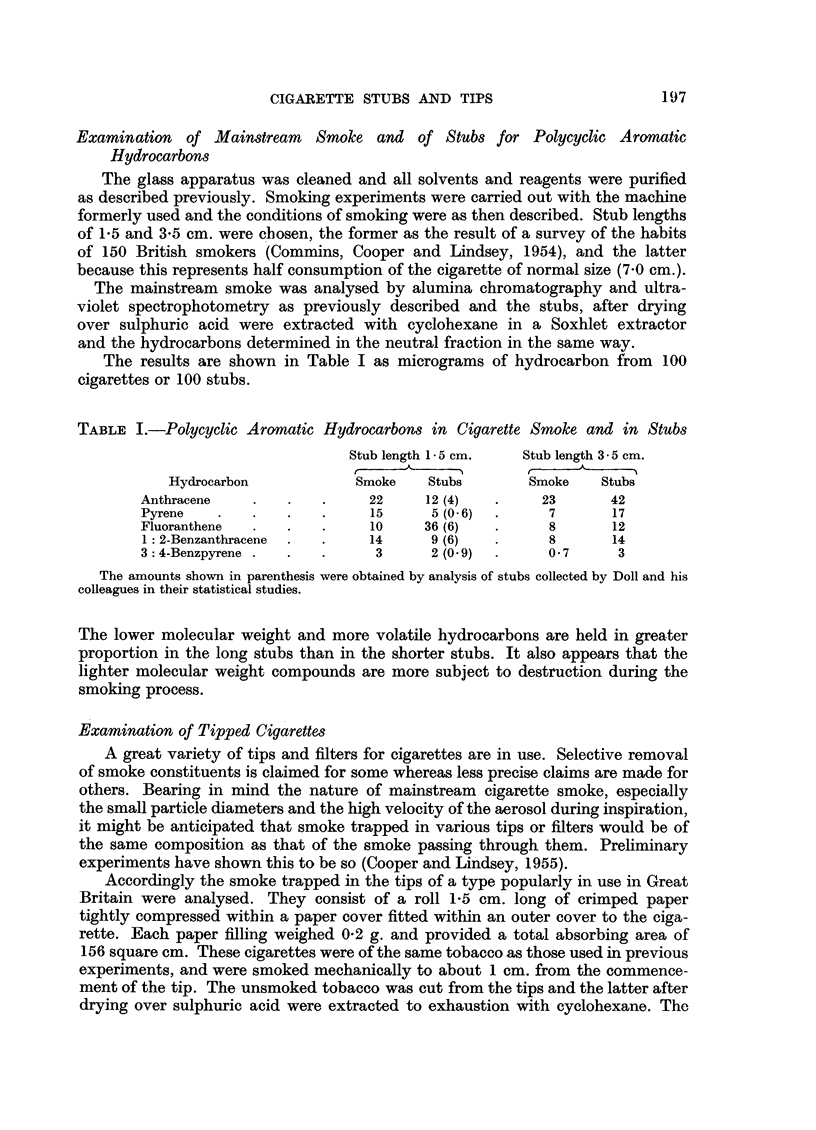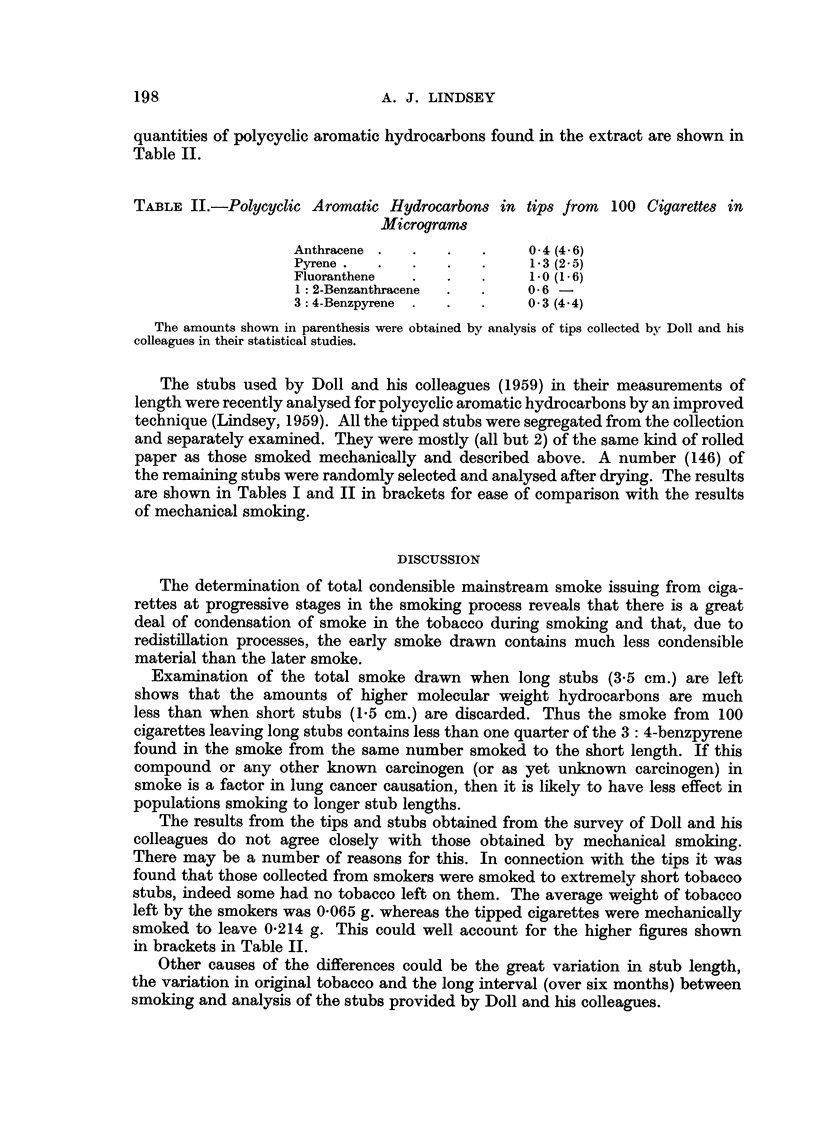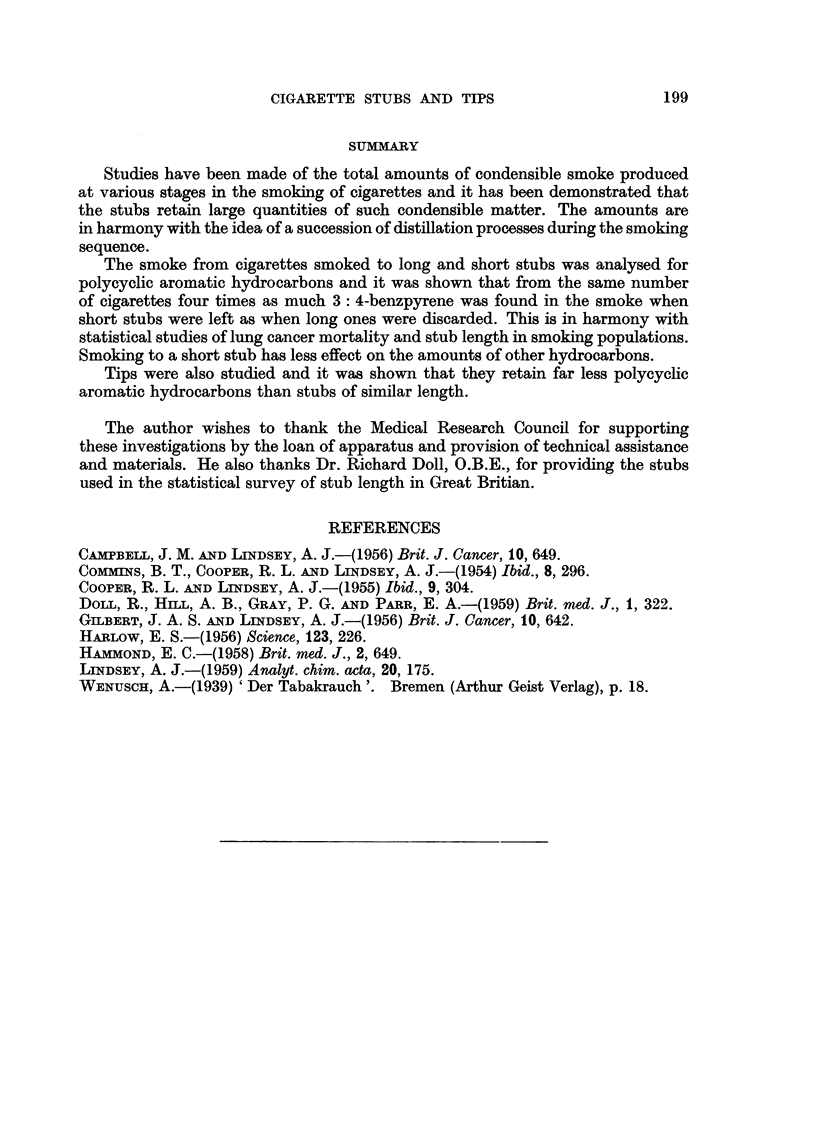# The Composition of Cigarette Smoke: Studies on Stubs and Tips

**DOI:** 10.1038/bjc.1959.26

**Published:** 1959-06

**Authors:** A. J. Lindsey


### THE COMPOSITION OF CIGARETTE SMOKE: STUDIES ON STUBS AND TIPS

A. J. LINDSEY

*From the Department of Chemistry, Sir John Cass College, London, E.C.3*

Received for publication March 18, 1959

IN earlier publications studies were made of the amounts of a number of polycyclic aromatic hydrocarbons in mainstream cigarette smoke (Cooper and Lindsey, 1955) and in the residual stubs and ash (Gilbert and Lindsey, 1956). These quantities showed that, after allowing for the amounts present in the unsmoked material, (Campbell and Lindsey, 1956) considerable quantities were trapped in the stubs and very small amounts were found in the ash. From these and other studies a mechanism for the smoking process was proposed (Gilbert and Lindsey, 1956) elaborating previous suggestions such as those of Wenusch (1939). In the present paper, studies of the mainstream smoke with varying stub length give additional support to these proposals and are also shown to be in harmony with statistical studies in U.S.A. (Hammond, 1958) and in Great Britain (Doll, Hill, Gray and Parr, 1959) on the relationship between lung cancer mortality and stub length.

The amounts of condensible compounds in mainstream cigarette smoke vary as the process goes on and are smaller at the commencement and heavier towards the end of a cigarette. It is therefore important to examine the composition of the inspired smoke at various stages during the smoking process and to relate this with the proposed mechanism of smoke formation.

*Outline of the smoking process*

The glowing zone of a cigarette during intervals between puffs is at about 650° C. and varies very little in temperature throughout its volume. During the suction periods the temperature rises by about 50° C. although superficial temperatures may be well over 900° C. It has also been shown that the temperature gradient behind the glowing zone is very steep; about 600° in 4 mm. (Harlow, 1956). During the quiescent or smouldering process, in a short region behind the glowing zone, distillation of various volatile constituents including water occurs and the products condense a little further back. In the region nearer the glowing zone thermal decomposition of the relatively non-volatile components occurs and some of the products of this process distil into the cooler regions while another portion emerges from the glowing zone in the side-stream smoke. In particular, amides and proteins give rise at this stage to ammonia and this is mainly responsible for the alkaline nature of the side stream smoke although the mainstream smoke may be acidic because of the excess of acidic constituents in it.

During suction, combustion is rapid and the distillation and thermal decomposition processes also occur much more quickly. The products, hot gases and steam from the combustion with extra steam from volatilized water, effect rapid steam distillation from the adjacent zones and the " smoke ", an aerosol consisting of droplets of a few microns in diameter, is quickly drawn through. Some of the aerosol condenses throughout the whole length of the cigarette. The repetitive distillation of products into a cool region near the glowing zone during quiescent periods and the more rapid distillation and steam distillation during suction produces a certain amount of fractionation so that the condensed material at later stages in the smoking process is richer in less volatile materials than at earlier stages. Thus, during later stages, there is more thermal decomposition of distilled materials with higher boiling points than occurs in earlier stages.

This outline of the smoking process although based upon existing knowledge is in part conjectural. It is now additionally supported by the following studies.

### EXPERIMENTAL

### *Determination of rate of production of condensible material in main-stream smoke*

Cigarettes with the desired stub length marked on the paper were smoked mechanically one by one in an apparatus similar to, but smaller than, that used

FIG. 1.—Condensible matter in mainstream cigarette smoke measured for various stub lengths. Amount from 1·5 cm. stub = 1.

previously (Cooper and Lindsey, 1955). The smoke was condensed in acetone which absorbed most of it ; the rest was allowed to settle and then the whole was transferred quantitatively with additional solvent into a graduated flask. The total amount of condensible matter obtained upon smoking a cigarette to a definite stub length was found by examining an aliquot portion of the acetone solution spectrophotometrically. The whole experiment was repeated several times and the average quantities are plotted in Fig. 1, whence it is seen that in the smoke from short stubs much greater quantities of condensible matter are present than from long stubs.

*Examination of Mainstream Smoke and of Stubs for Polycyclic Aromatic Hydrocarbons*

The glass apparatus was cleaned and all solvents and reagents were purified as described previously. Smoking experiments were carried out with the machine formerly used and the conditions of smoking were as then described. Stub lengths of 1·5 and 3·5 cm. were chosen, the former as the result of a survey of the habits of 150 British smokers (Commins, Cooper and Lindsey, 1954), and the latter because this represents half consumption of the cigarette of normal size (7·0 cm.).

The mainstream smoke was analysed by alumina chromatography and ultra-violet spectrophotometry as previously described and the stubs, after drying over sulphuric acid were extracted with cyclohexane in a Soxhlet extractor and the hydrocarbons determined in the neutral fraction in the same way.

The results are shown in Table I as micrograms of hydrocarbon from 100 cigarettes or 100 stubs.

TABLE I.—*Polycyclic Aromatic Hydrocarbons in Cigarette Smoke and in Stubs*

| | Stub length 1·5 cm. | | Stub length 3·5 cm. | |
|---|---|---|---|---|
| Hydrocarbon | Smoke | Stubs | Smoke | Stubs |
| Anthracene | 22 | 12 (4) | 23 | 42 |
| Pyrene | 15 | 5 (0·6) | 7 | 17 |
| Fluoranthene | 10 | 36 (6) | 8 | 12 |
| 1 : 2-Benzanthracene | 14 | 9 (6) | 8 | 14 |
| 3 : 4-Benzpyrene | 3 | 2 (0·9) | 0·7 | 3 |

The amounts shown in parenthesis were obtained by analysis of stubs collected by Doll and his colleagues in their statistical studies.

The lower molecular weight and more volatile hydrocarbons are held in greater proportion in the long stubs than in the shorter stubs. It also appears that the lighter molecular weight compounds are more subject to destruction during the smoking process.

*Examination of Tipped Cigarettes*

A great variety of tips and filters for cigarettes are in use. Selective removal of smoke constituents is claimed for some whereas less precise claims are made for others. Bearing in mind the nature of mainstream cigarette smoke, especially the small particle diameters and the high velocity of the aerosol during inspiration, it might be anticipated that smoke trapped in various tips or filters would be of the same composition as that of the smoke passing through them. Preliminary experiments have shown this to be so (Cooper and Lindsey, 1955).

Accordingly the smoke trapped in the tips of a type popularly in use in Great Britain were analysed. They consist of a roll 1·5 cm. long of crimped paper tightly compressed within a paper cover fitted within an outer cover to the cigarette. Each paper filling weighed 0·2 g. and provided a total absorbing area of 156 square cm. These cigarettes were of the same tobacco as those used in previous experiments, and were smoked mechanically to about 1 cm. from the commencement of the tip. The unsmoked tobacco was cut from the tips and the latter after drying over sulphuric acid were extracted to exhaustion with cyclohexane. The quantities of polycyclic aromatic hydrocarbons found in the extract are shown in Table II.

TABLE II.—*Polycyclic Aromatic Hydrocarbons in tips from* 100 *Cigarettes in Micrograms*

| | |
|---|---|
| Anthracene | 0·4 (4·6) |
| Pyrene | 1·3 (2·5) |
| Fluoranthene | 1·0 (1·6) |
| 1 : 2-Benzanthracene | 0·6 — |
| 3 : 4-Benzpyrene | 0·3 (4·4) |

The amounts shown in parenthesis were obtained by analysis of tips collected by Doll and his colleagues in their statistical studies.

The stubs used by Doll and his colleagues (1959) in their measurements of length were recently analysed for polycyclic aromatic hydrocarbons by an improved technique (Lindsey, 1959). All the tipped stubs were segregated from the collection and separately examined. They were mostly (all but 2) of the same kind of rolled paper as those smoked mechanically and described above. A number (146) of the remaining stubs were randomly selected and analysed after drying. The results are shown in Tables I and II in brackets for ease of comparison with the results of mechanical smoking.

### DISCUSSION

The determination of total condensible mainstream smoke issuing from cigarettes at progressive stages in the smoking process reveals that there is a great deal of condensation of smoke in the tobacco during smoking and that, due to redistillation processes, the early smoke drawn contains much less condensible material than the later smoke.

Examination of the total smoke drawn when long stubs (3·5 cm.) are left shows that the amounts of higher molecular weight hydrocarbons are much less than when short stubs (1·5 cm.) are discarded. Thus the smoke from 100 cigarettes leaving long stubs contains less than one quarter of the 3 : 4-benzpyrene found in the smoke from the same number smoked to the short length. If this compound or any other known carcinogen (or as yet unknown carcinogen) in smoke is a factor in lung cancer causation, then it is likely to have less effect in populations smoking to longer stub lengths.

The results from the tips and stubs obtained from the survey of Doll and his colleagues do not agree closely with those obtained by mechanical smoking. There may be a number of reasons for this. In connection with the tips it was found that those collected from smokers were smoked to extremely short tobacco stubs, indeed some had no tobacco left on them. The average weight of tobacco left by the smokers was 0·065 g. whereas the tipped cigarettes were mechanically smoked to leave 0·214 g. This could well account for the higher figures shown in brackets in Table II.

Other causes of the differences could be the great variation in stub length, the variation in original tobacco and the long interval (over six months) between smoking and analysis of the stubs provided by Doll and his colleagues.

### SUMMARY

Studies have been made of the total amounts of condensible smoke produced at various stages in the smoking of cigarettes and it has been demonstrated that the stubs retain large quantities of such condensible matter. The amounts are in harmony with the idea of a succession of distillation processes during the smoking sequence.

The smoke from cigarettes smoked to long and short stubs was analysed for polycyclic aromatic hydrocarbons and it was shown that from the same number of cigarettes four times as much 3 : 4-benzpyrene was found in the smoke when short stubs were left as when long ones were discarded. This is in harmony with statistical studies of lung cancer mortality and stub length in smoking populations. Smoking to a short stub has less effect on the amounts of other hydrocarbons.

Tips were also studied and it was shown that they retain far less polycyclic aromatic hydrocarbons than stubs of similar length.

The author wishes to thank the Medical Research Council for supporting these investigations by the loan of apparatus and provision of technical assistance and materials. He also thanks Dr. Richard Doll, O.B.E., for providing the stubs used in the statistical survey of stub length in Great Britian.

### REFERENCES

CAMPBELL, J. M. AND LINDSEY, A. J.—(1956) *Brit. J. Cancer*, **10**, 649.
COMMINS, B. T., COOPER, R. L. AND LINDSEY, A. J.—(1954) *Ibid.*, **8**, 296.
COOPER, R. L. AND LINDSEY, A. J.—(1955) *Ibid.*, **9**, 304.
DOLL, R., HILL, A. B., GRAY, P. G. AND PARR, E. A.—(1959) *Brit. med. J.*, **1**, 322.
GILBERT, J. A. S. AND LINDSEY, A. J.—(1956) *Brit. J. Cancer*, **10**, 642.
HARLOW, E. S.—(1956) *Science*, **123**, 226.
HAMMOND, E. C.—(1958) *Brit. med. J.*, **2**, 649.
LINDSEY, A. J.—(1959) *Analyt. chim. acta*, **20**, 175.
WENUSCH, A.—(1939) ‘ Der Tabakrauch ’. Bremen (Arthur Geist Verlag), p. 18.